# Deciphering Driver of Nasopharyngeal Cancer Development

**DOI:** 10.3389/or.2022.10654

**Published:** 2022-10-11

**Authors:** Handoko, Melva Louisa, Tiara Bunga Mayang Permata, Soehartati A. Gondhowiardjo

**Affiliations:** ^1^ Doctoral Program in Biomedical Sciences, Faculty of Medicine, Universitas Indonesia, Depok, Indonesia; ^2^ Department of Radiation Oncology, Cipto Mangunkusumo National General Hospital, Jakarta, Indonesia; ^3^ Department of Pharmacology and Therapeutics, Faculty of Medicine, Universitas Indonesia, Depok, Indonesia; ^4^ Department of Radiation Oncology, Faculty of Medicine, Universitas Indonesia, Depok, Indonesia

**Keywords:** immunology, nasopharyngeal cancer, carcinogenesis, EBV, HLA polymorphism, MHC

## Abstract

A great deal of progress has been made on understanding nasopharyngeal cancer in recent decades. Genomic, transcriptomic, and proteomic studies have enabled us to gain a deeper understanding on the biology of nasopharyngeal cancer, and though this new information is elaborate and detailed, an overall picture of the driver of nasopharyngeal cancer that includes all this information is lacking. This review will focus on providing a broad overview, with plausible and simple language, on nasopharyngeal carcinogenesis based on current updated information. This will help readers to gain a broad understanding, which may be necessary to provide common ground for further research on nasopharyngeal cancer.

## Introduction

Nasopharyngeal cancer is an endemic cancer in some parts of the world including Southern and South East Asia (1, 2). However, this is a very rare cancer in the western part of the world (3). Based on Global Cancer Observatory data, incidences of nasopharyngeal cancer made up only 0.16% of all cancers in 2020, with more than 85% of the incidences occurring in Asia. Treatment outcome has improved in the past decade for those without metastatic disease. This was due to the improvement of radiotherapy technique and technology. As radiotherapy is the mainstay treatment in non-metastatic nasopharyngeal cancer, advancement in its technology has enabled a greater dose to be delivered to the tumour while reducing the effect on the normal organs in the surrounding area. Nevertheless, around 15%–50% of patients would eventually relapse (4, 5). Those with relapsed disease or metastatic disease on diagnosis or those without complete tumour response after primary definitive chemoradiation treatment would have much poorer prognoses.

A lot of efforts have been made to improve the outcome of those patients with relapse and unsatisfactory treatment response after primary definitive chemoradiation (6, 7). In the clinical realm, the intensification of treatment by adding more chemotherapy before definitive chemoradiation has been attempted with somewhat marginal benefit at a cost of more toxicities (6, 7). Another approach was to add adjuvant chemotherapy after definitive chemoradiation, though this approach still has not yielded a satisfactory outcome (8). Another approach put forward was to utilize immunotherapy such as PD-1 blockage antibodies. The recent KEYNOTE-122 results did not show survival advantage of patients with recurrent or metastatic nasopharyngeal cancer treated with either pembrolizumab (anti PD-1 immunotherapy) or standard chemotherapy (9).

All of these unsatisfactory results in attempting to improve treatment outcomes in those subpopulations of nasopharyngeal cancer indicate that we need a different approach. A comprehensive understanding of tumour biology, its interaction with host cells, and the response of our immune system in deterring cancer is necessary. This knowledge will underpin a more straightforward strategy in attempts to improve outcomes for these subpopulations of patients. A lot is understood regarding nasopharyngeal cancer biology and its relationship with EBV infection, but still more questions arise. We now have multiple comprehensive genomic studies, transcriptomic studies, molecular studies, and clinical studies on nasopharyngeal cancer. Those studies have shed light on nasopharyngeal cancer, but a comprehensive and plausible understanding on nasopharyngeal cancer progression is not available. This review will combine all of this information and present it in a simple, plausible, and understandable manner. This is important offer updated knowledge that can serve as a foothold for further nasopharyngeal cancer studies.

## Nasopharyngeal Cancer and Epstein Barr Virus Status

Epstein Barr virus (EBV) infection is an endemic infection worldwide. Normally, in humans, EBV commonly infects B cells, in which the majority of cases are asymptomatic or mildly symptomatic (10). This is a self-limiting disease. However, in a small percentage of individuals, it may later develop into malignancy, typically present as lymphoid or epithelial malignancy (11). After an active EBV infection, the virus can switch modes to dormant or latent infection, in which the viruses reside within B cells or epithelial cells.

In latent EBV infection, the viruses express various latent proteins which enable the viruses to maintain presence in the host cells undetected from our immune system. Limited EBV genes are expressed such as latent membrane protein 1 (LMP1), latent membrane protein 2 (LMP2), EBV-encoded nuclear antigen-1 (EBNA1), EBV-encoded nuclear antigen-2 (EBNA2), EBV-encoded nuclear antigen-3 (EBNA3), EBV-encoded small RNA (EBER), non-transcribed BamHI-A region rightward transcript (BART) RNAs, etc. (12) All of those EBV latent genes function to maintain EBV presence in host cells and ensure the continuous survival and proliferation of the infected host cells (13).

The most common nasopharyngeal cancer is the undifferentiated type of nasopharyngeal cancer. This type of nasopharyngeal cancer is always associated with (EBV) infection and has a predisposition to occur in certain populations only. Typically, it is very common in South Asia, South East Asia, North Africa, and Middle East populations (1). Multiple genome wide studies have identified various polymorphisms in human leukocyte antigen (HLA) genes to be strongly associated with a risk of nasopharyngeal cancer development (14). HLA genes are highly diverse genes that have an important function in recognizing antigens and are involved in mounting immune response. Polymorphism is a normal evolutionary process, to ensure a genetic diversity that will be beneficial for our continuous survival as a species. However, due to its random process, there is a possibility that a certain haplotype becomes prone to developing nasopharyngeal cancer (15).

Genome wide analysis has revealed multiple important copy numbers and structural aberrations from clinical specimens of nasopharyngeal cancer (16). Loss of important segments of genes that code for pro-inflammatory cytokines type 1 interferon such as IFNA1, IFNA2, IFNA8, and IFNE was noted (16). This loss of IFN might result in blunted immunological response upon primary EBV infection. Furthermore, without sufficient IFN in the tumour microenvironment, there would be a possibility of a reduced capacity of CD8 T cells in mounting an immunological attack toward tumour cells. Homozygous loss of important cell cycle controllers was also noted. CDKN2A/CDKN2B were the most commonly deleted genes (16). These genes were responsible for keeping cell cycles in check, thus, loss of these genes promote the development of cancer.

EBV latent oncoproteins were also found in nasopharyngeal cancer to further stimulate the niche for tumour development. LMP1 was found to exert its activity through the activation of several pathways in the host cells including NF-κB, JNK–p-38, PI3K–AKT, ERK–MAPK, and JAK–STAT (16, 17). Activation of those pathways were known to stimulate various transcription factors that ultimately promote cell survival. LMP1 was also implicated in causing promoter hypermethylation through DNA methyltransferase 1 (DNMT1) (17), resulting in a greater chance of inactivating expression of important tumour suppressor genes. All of these effects synergistically with inherent host genomic alterations promote cancer development.

## Tumour Microenvironment in Nasopharyngeal Cancer

The tumour microenvironment has been shown to have an increasingly important role in tumour development and the failure of various cancer treatments (18). The tumour microenvironment consists of various cells including malignant cells, immune cells, and various stromal cells interacting and determining the growth and death of those cells. Understanding the interaction of those cells within tumour microenvironment is critical to the ability to develop a strategy to tip the balance to favour tumour death.

Within nasopharyngeal cancer cells of the same patient, there is a tremendous amount of heterogeneity among tumour cells (19). A recent RNA sequencing of single cells resolution has confirmed that not all malignant cells were EBV positive (19). Those cells with EBV were found to have a distinct expression profile, typically with enrichment of cytokine expression, regulation of cell death, apoptosis, and cancer-related pathways (19). Those expressions were thought to confer an increased susceptibility toward EBV infection. However, a closer look at other expression profiles found that even among those with EBV positive malignant cells, there was a very heterogenous expression profile indicating numerous malignant cell clones within a single patient (19). These multiple malignant cell clones would confer benefit for tumour survival but could complicate an effective treatment that can lead to a cure.

In nasopharyngeal cancer, a particular pattern of the tumour microenvironment has been shown to favour a better prognosis. Typically, a microenvironment with dense immune cells infiltrate was associated with a less aggressive tumour (20, 21). Immune cells infiltrate, particularly CD8 cytotoxic T cells, are the main effector cells that attack cancer cells. Thus, it is plausible that denser CD8 T cells infiltrate within the tumour microenvironment is a positive sign. Nevertheless, even in a dense tumour microenvironment, eventually cancer still developed. This fact indicated that some sort of mechanistic failure on immune attack was happening. The following section will discuss the current evidence that explains the possibility of a failed immune attack.

## Functionality of Immune Cells in Tumour Microenvironment of Nasopharyngeal Cancer

Nasopharyngeal cancer generally has much denser immune cells infiltrates within its microenvironment compared to other solid cancers (19). Traditionally, this cancer was known as lymphoepithelial carcinoma because of its highly characteristic lymphocyte infiltrates. In immune-oncology, this kind of tumour with such a characteristic microenvironment is called a hot tumour. Generally, compared to other types of tumour a hot tumour is considered to be a more favourable tumour, which theoretically responds well to simple immunotherapy such as anti-check point blockage. This nasopharyngeal cancer generally has also high expression of PD-L1 (21), which further suggests that such immunotherapy might work. Nevertheless, well designed clinical trials such as KEYNOTE-122 have failed to prove the benefit of immunotherapy in metastatic nasopharyngeal cancer (9).

The assessment of the nasopharyngeal cancer microenvironment on a cellular level has revealed a very heterogenous immune cell population with different phenotypes (19, 22). In a comprehensive single cell RNA analysis, based on a differentially expressed gene pattern, it was found that the majority of CD 8 T cells in the tumour microenvironment were expressing a marker of T cell exhaustion (19, 22). Those exhausted CD8 T cells were also actively proliferating, shown by multiple clonotypes and cell clones based on T cell receptor analysis (19). On trajectory analysis, these exhausted T cells were found to be at the terminally differentiated state (19). This meant that these CD8 T cells were induced from their naive state to exhausted state, and along this process these cells became more terminally differentiated.

Furthermore, tolerogenic cells such as T regulatory (T reg) cells and myeloid-derived suppressor cells (MDSCs) were also found to be very common in the nasopharyngeal cancer microenvironment (19, 22). These T reg cells were found to express high IL2 receptors, immune inhibitory markers, and chemotactic markers (19). These patterns of expression indicated that these T reg cells were highly active, communicating and possibly recruiting other immune tolerance cells, altogether resulting in a very immune suppressive environment. This partially explained the finding of very exhaustive CD8 T cells that failed to eliminate tumour cells.

Dendritic cells, an important type of residential cell, which are part of innate immune response with a role in eliciting initial antigen presentation were also found to be exhaustive in the nasopharyngeal cancer TME (19). In the single cell RNA sequencing study, multiple clusters of dendritic cells with distinct expression patterns were found. The cluster of dendritic cells with high expression of LAMP3 was found to be highly active, with high migratory potential and maturation markers. These LAMP3 dendritic cells among other clusters of dendritic cells were found to have the lowest capacity in antigen presentation but the highest expression of immune regulatory markers such as CD274 (PD-L1), PDCD1LG2 (PD-L2), CD200, EBI3, IDO1, IL4I1, SOCS1, SOCS2, and SOCS (19).

Other clusters of dendritic cells showed a capacity for antigen presentation (19), which is normally expected for dendritic cells. However, further trajectory analysis suggested the dendritic cells with intact antigen presentation potential were naive or less mature, while the LAMP3 dendritic cells were highly mature (19). Overall, these findings meant that dendritic cells initially had proper capacity for antigen presentation, carried out their tasks, but later on were induced to become more differentiated with shift of expression pattern to be more tolerogenic and dull on antigen presentation capacity.

## Plausible Intercellular Interaction in Nasopharyngeal Cancer Microenvironment that Led to Cancer Progression

**FIGURE 1:**
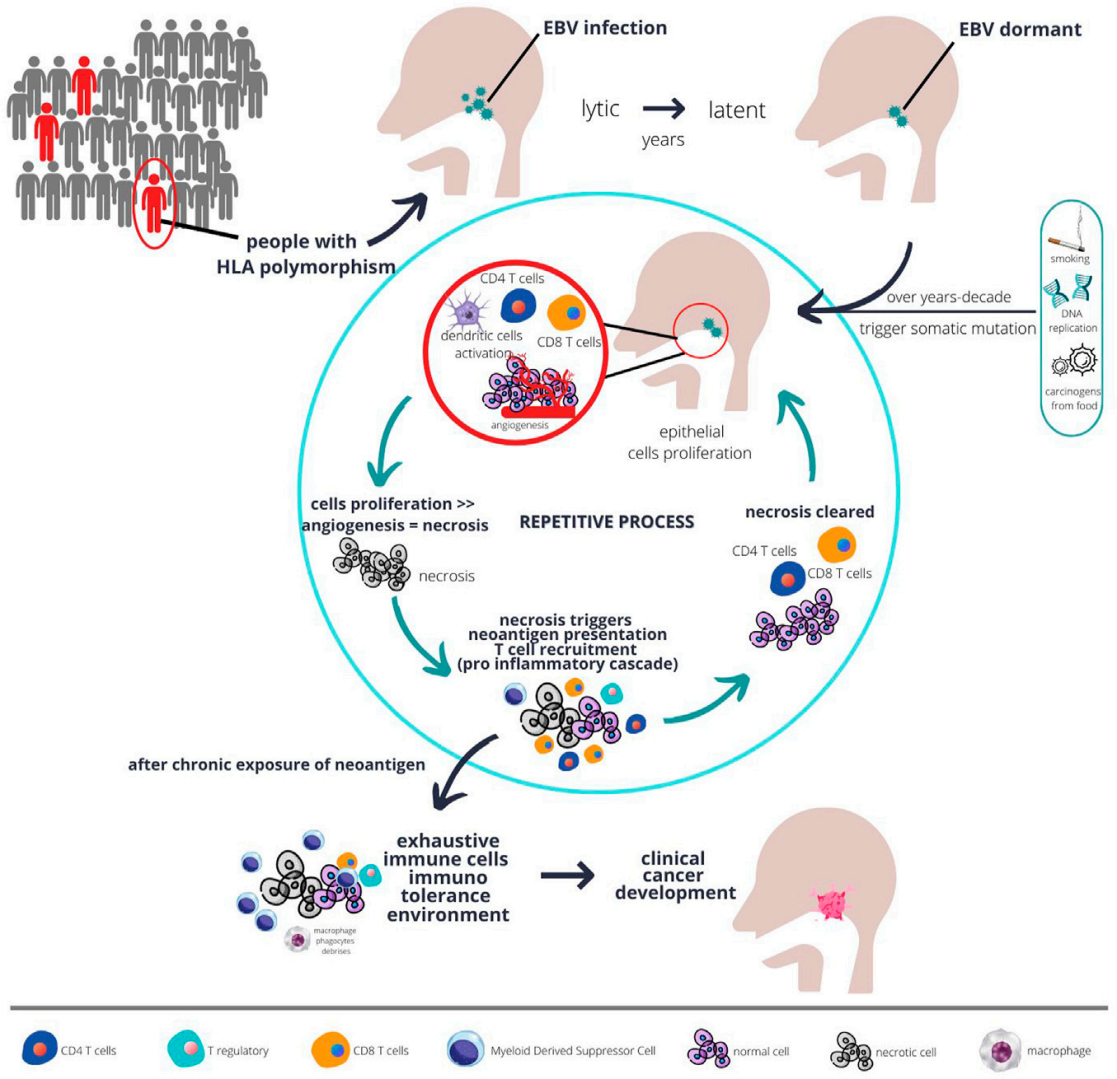
The plausible interaction between host genetics, EBV, immunity, and carcinogens over a long period of time result in development of fulminant nasopharyngeal cancer. Populations at risk, such as those with certain type of HLA polymorphism, when infected with chronic EBV together with environmental risk factors are more likely to develop abnormal epithelial proliferation. Chronic proliferations coupled with sluggish angiogenesis result in necrosis, which in turn triggers immune response. However, with chronic inflammation, over time the immune system develops tolerance and proliferation eventually unopposed, and cancer starts to develop.

The nasopharyngeal cancer microenvironment contains very heterogenous cells with most immune cells being dysfunctional, but highly proliferative. However, those immune cells are not the cause of cancer development, *per se*. The EBV infection in initially vulnerable individuals, which has a particular haplotype, triggers the commencement of EBV latent infection. These EBV latent genes promote the activation of several pathways as described earlier, together with somatic mutation in epithelial cells, resulting in the development of the very first clone of a malignant cell (see Figure 1).

Due to the low mutational rate in nasopharyngeal cancer, but high epigenetic changes particularly hypermethylation, the error in host genetics is thought to be unlikely to trigger immune response. Immune response is initially triggered by the EBV infection in the lytic phase. However, some EBV infection may switch modes to the latent phase and remain dormant within the cells. Overtime, with increasing exposure to environmental carcinogens or accumulated error in DNA replications that goes unrepaired due to somatic mutations (as described in the previous section), those cells start to gain more potential for uncontrolled proliferation. The tumour will proliferate at a fast pace with some parts possibly becoming necrotic due to the imbalance between angiogenesis and tumour growth (see Figure 1). This is evidenced by the necrotic area seen on a histopathology examination of nasopharyngeal cancer (23).

The tumours subsequently become more immunogenic. The necrosis results in a release of neoantigens from both tumour mutation and EBV antigen which is readily phagocyted by residential dendritic cells. These initial naive dendritic cells will complete the task of antigen presentation to CD4 T cells, secreting cytokines and chemokines to further recruit more T cells. The culmination of this pro-inflammatory immunological response will recruit lots of CD8 T cells to the TME. Those CD8 T cells will initially react and become effector cells to clear any tumours that express neoantigens.

Due to the highly heterogenous nature of the tumour, not all tumours are able to express tumour neoantigens. Those tumours with loss of function in genes such as HLA-A, NLRC5 will lose the ability to present tumour neoantigens to dendritic cells or CD4 T cells (16, 24). This HLA-A gene and transcription activator NLRC5 are the fundamental machinery for antigen presentation of non-professional antigen presentation cells. Thus, this kind of tumour cell will not be attacked by CD8 T cells because they are not tagged as harbouring tumour neoantigens. These clones of tumour cells will continue to proliferate until a point where necrosis occur again due to the imbalanced supply of blood vessels vs. tumour growth.

With continual cycles of necrosis happening intermittently, those initial dendritic cells will be exposed to cyclical neoantigens. Physiologically, with continuous chronic exposure of neoantigens, the pro-inflammatory immune response will be blunted (20). Dendritic cells, CD4 T cells, and CD8 T cells will undergo maturation or differentiation due to the continual presence of tumour neoantigens. Along the process of differentiation, those cells will also become dysfunctional (see Figure 1), proven by the high expression of exhaustive markers, as elaborated upon in the previous section.

The process of immune cells exhaustion is mediated by various cytokines and their corresponding receptors. Dendritic cells interacted with T reg cells through CCL17-CCR4 and CCL22-CCR4, resulting in the recruitment of T reg cells into the TME (19). Dendritic cells also interacted with CD8 T cells through CD200 - CD200R signalling pathway, resulting in a blunting of the immune response (19, 25). T reg cells, dendritic cells, and CD8 T cells were shown to also interact through CTLA4 and CD80/CD86 ligand receptors interactions, in which this possible interaction was further strengthened by the fact that those cells were juxtaposed in a multiplex immunohistochemistry study (19). These prominent interactions were detected from an RNA sequencing study, but a more meticulous study that is specifically designed to study cellular interaction is necessary to completely understand the crosstalk mechanism between cells in the nasopharyngeal cancer TME.

Through understanding the cellular interactions within the nasopharyngeal cancer TME, we can propose various possible therapeutic approaches. For instance when the dominating mechanism of a particular nasopharyngeal cancer patient is the highly exhaustive TME, then we can propose an anti T reg immunotherapy (2, 26). However, the main constraint here is that describing the TME of a particular patient is very challenging because it requires an iterative individualised method and there is no particular workflow that can fit for all patients. Nevertheless, with the advancement of computing power and artificial intelligence, an individualised approach in describing the TME and then proposing a therapeutic approach may be possible in the future.

## Conclusion

Typical haplotypes in at risk populations in nasopharyngeal cancer endemic regions predispose someone to malignant cells development. Further, EBV infection in the nasopharynx epithelium kick-starts the driver of those cancer hallmarks. Cancer cell development is bolstered with further somatic mutation due to environmental exposure of carcinogens or inherent DNA damage from replication errors. With excessive proliferation of cancer cells, necrosis ensues, which results in the release of neoantigens. Immune cells kick in to restrain the development of cancer. However, due to the heterogeneity of the cancer cells, eventually, some clones can escape immune recognition. Further chronic sensitization of neoantigens from the tumour cause the local milieu to favour a tolerogenic immune environment. This results in the suppression of the cancer immune attack and unrestrained cancer progression.
